# Disinformation on dietary supplements by German influencers on Instagram

**DOI:** 10.1007/s00210-024-03616-4

**Published:** 2024-11-25

**Authors:** Jan-Niklas Ricke, Roland Seifert

**Affiliations:** Institute of Pharmacology, Hannover Medical School, Carl-Neuberg-Str. 1, D-30655 Hanover, Germany

**Keywords:** Dietary supplements, Overdose, UL, Vitamins, Minerals, Social media, Instagram, German influencers

## Abstract

A significant proportion of the German population regularly consumes dietary supplements (NEM), and the market for these products is growing steadily. Dietary supplements are subject to food law and not pharmaceutical law. There are no official limits for the dosages of vitamins and minerals in Germany—only recommendations from institutions such as the Bundesinstitut für Risikobewertung (BfR) or the European Food Safety Authority (EFSA). Dietary supplements are strongly advertised on Instagram in particular. In Germany, Instagram is the most popular social network and many influencers use the platform to promote dietary supplements. In this paper, the ingredients of 105 dietary supplements promoted by German influencers on Instagram from 2021 to 2023 were analyzed. This analysis was based on various parameters, such as dosage form, daily therapy costs, overdose warnings, presence of dosage information, exceeding the recommended maximum daily amounts and tolerable upper intake levels (UL) for vitamins and minerals and information on adverse effects, drug interactions, and contraindications. About two-thirds of the intensively advertised dietary supplements exceeded the recommended maximum daily amounts of the Bundesinstitut für Risikobewertung (BfR) for vitamins and minerals without the influencers pointing out the negative effects of an overdose. Dietary supplements were frequently advertised on Instagram with discount codes, promising supplement names and promises of effectiveness and were often presented as a panacea. In contrast, information on dosing, daily costs, adverse effects, contraindications, and risks of overdosing were insufficiently addressed by influencers. Overall, influencers on Instagram disinform rather than inform consumers on dietary supplements, opening the door for intoxications. Therefore, legal action is required to prevent disinformation by influencers on social media.

**Supplementary Information:**

The online version contains supplementary material available at 10.1007/s00210-024-03616-4.

## Introduction

Dietary supplements are popular in the German population. According to a survey conducted by the Bundesinstitut für Risikobewertung (BfR) in 2021, one in three respondents took a dietary supplement at least once a week (BfR 2021). According to another survey from 2022, three out of four respondents had taken a dietary supplement in the last 12 months - primarily vitamins (61%) and minerals (36%) (Statista Daily Data, Brandt 2023). According to the BfR-Verbrauchermonitor, Germans primarily take vitamin D, vitamin B12, vitamin C, and multivitamin supplements (BfR 2021).

**Fig. 1:**
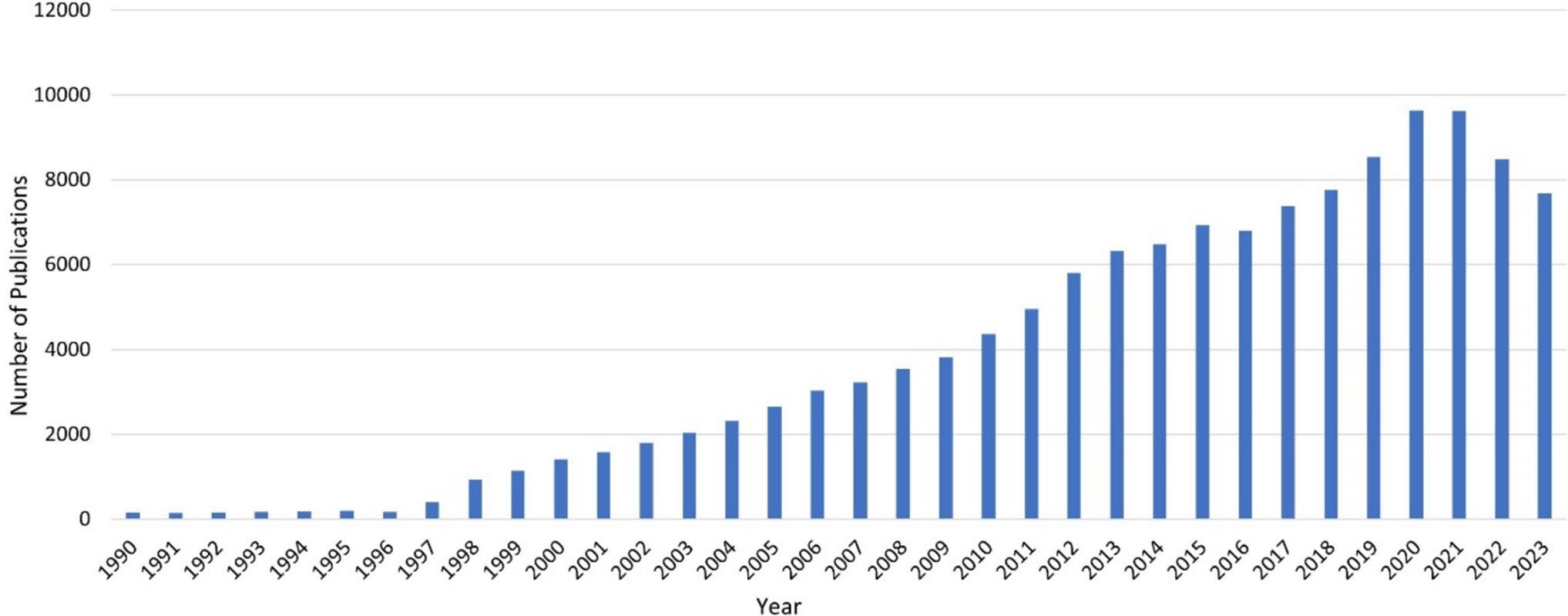
Chronological development of publications for the search terms “dietary supplements” (all fields) on PubMed.gov (retrieved on 30.04.2024)

In 2022, three billion euros were spent on dietary supplements in German pharmacies alone (Statista Daily Data, Neumann 2022). The relevance and actuality of the topic are also evident in the literature. Figure 1 shows the increasing number of publications on the topic of “dietary supplements” on PubMed.

Dietary supplements are not medicinal products and therefore do not fall under the Arzneimittelgesetz (AMG). They are foodstuffs and are subject to food law (Lebensmittel- und Futtermittelgesetzbuc-LFGB and Verordnung über Nahrungsergänzungsmittel-NemV). This means that dietary supplements are freely available for sale and accessible to everyone. In contrast to medicinal products, dietary supplements are not subject to testing or approval by an authority. The manufacturer itself is responsible for the dosage of the ingredients and the health safety of dietary supplements (Bundesamt für Verbraucherschutz und Lebensmittelsicherheit (BVL)).

There are no fixed maximum amounts for the ingredients (vitamins, minerals, amino acids, etc.) in dietary supplements—only recommendations from institutions such as the Bundesinstitut für Risikobewertung (BfR), the European Food Safety Authority (EFSA), or the Deutsche Gesellschaft für Ernährung (DGE). However, these recommendations are not legally binding (BVL). There is therefore a considerable risk of overdosing and potential toxic damage (Rathmann and Seifert 2024).

Instagram is a widely used social networking service for sharing photos and videos and belongs to Meta Platforms, the parent company of Facebook, Threads, WhatsApp, Messenger, and Meta Quest. Instagram was launched on October 6, 2010 by co-founders Kevin Systrom and Mike Krieger and quickly reached significant user numbers. The platform is available in 32 languages and supports a variety of functions such as posts, stories and direct messaging.

By 2022, Instagram has surpassed the two billion monthly active users mark, making it one of the largest social media platforms in the world. The user base and engagement metrics have attracted many advertisers and helped to popularize influencer marketing. This growth underlines Instagram's major influence on the digital and social media landscape (Encyclopedia Britannica, Eldridge 2024). The largest user base worldwide in 2022 was the 25–34 age group (Statista Daily Data, Statista Research Department 2024).

In July 2024, around 33.8 million Germans use the social network Instagram. The largest user group is also made up of 25 to 34-year-olds (Statista Daily Data, Dixon 2024). According to an online survey conducted by ARD and ZDF in 2023, Instagram is the most popular social network in Germany, ahead of Facebook (Media Perspektiven, Koch 2023).

Young people in particular use the platform. It is this young user base that is increasingly obtaining health information via social media (Nath et al. 2024; Pilgrim and Bohnet-Joschko 2019; Zamil et al. 2022). Pilgrim and Bohnet-Joschko analyzed 20 posts from each of the 50 most popular fitness and lifestyle influencers from Germany using the hashtags “gym,” “fit,” “fitness,” “sport,” “nutrition,” “train,” and “food” according to the content of the posts, the influencers’ communication strategies and the impact on users. The study showed that influencers gain the trust and sympathy of their followers through body-oriented content and targeted communication. They promote diet and exercise as controllable factors for body perfection and advertise dietary supplements and tight branded sportswear as simple methods for optimizing appearance. This leads to followers becoming dependent on the influencers (Pilgrim and Bohnet-Joschko 2019).

The dietary supplement market is constantly expanding and social media users are exposed to a wide range of dietary supplements. Nevertheless, 82% of the people surveyed by the BfR in 2021 stated that they had not been diagnosed with a vitamin deficiency. Likewise, 78% of respondents rated the health risk from vitamins as dietary supplements as “medium” or “(very) low.” At the same time, 70% of respondents felt “moderately” or “(not at all) well” informed about the health risk of vitamins as dietary supplements (BfR 2021).

The toxicity of some vitamins and minerals when taken in high doses has been described in the literature, e.g. hepatotoxicity (Biesalski 1989; Hathcock et al. 1990; Nollevaux et al. 2006) and teratogenicity (Hayes et al. 1981; Hendrickx et al. 2000; Rothman et al. 1995) due to vitamin A or peripheral polyneuropathy with vitamin B6 (Bossard et al. 2022, Moses 2021). The occurrence of hypercalcemia because of chronic excessive vitamin D supplementation can lead to the formation of kidney stones as well as calcium deposits in the tissue (Janoušek et al. 2022; Rizzoli 2021). In addition, there is an increased risk of fractures (Moses 2021; Pilz et al. 2018; Rizzoli 2021) and an increased risk of falls, especially in older people (Pilz et al. 2018; Rizzoli 2021).

An overdose of magnesium leads to hypermagnesemia, which can cause nausea, vomiting, stomach cramps, and flushing, among other things (Moses 2021). Similar vegetative symptoms are to be expected with an overdose of zinc - nausea, vomiting, abdominal cramps, diarrhea and headaches (EFSA 2006, Moses 2021). It is therefore not advisable to take dietary supplements containing vitamins and minerals at random.

In contrast to other studies, which primarily deal with the product information of dietary supplements, communication strategies and advertising strategies on social media (s. Basch et al. 2016; Denniss et al. 2023;Nath et al. 2024; Pilgrim and Bohnet-Joschko 2019; Zamil et al. 2022), this study is the first to analyze the individual active ingredients and their dosages of the dietary supplements advertised by German influencers. The dosages of vitamins and minerals are examined using scientifically based reference values. These include the maximum daily intake recommendations of the BfR, the daily reference intake recommendations of the BfR and Verordnung (EU) Nr. 1169/2011, and the upper intake levels (UL) published by the EFSA. Particular attention is paid to preparations whose active ingredients exceed the reference values. The possible toxic effects of the overdosed vitamins and minerals are discussed based on the existing literature.

## Materials and methods

All information about the NEM on Instagram can be found in the influencers’ posts. These posts are accessible to everyone, as only public accounts were analyzed. Further criteria for narrowing down the search for posts were as follows: only German influencers; influencers with more than 10,000 followers; posts from 2021 to 2023; no brand or manufacturer pages and no videos or reels. The posts were found using the hashtags “nahrungsergänzungsmittel,” “nahrungsergänzung,” and “influencer.” The search function on Instagram was used for the search: https://www.instagram.com/.

The manufacturer information on the dietary supplements found on Instagram was researched on the respective manufacturer’s website. Where necessary, the packaging of the dietary supplements was also consulted.

The final sample comprised 105 NEM from 78 Instagram posts by 61 different influencers (51 female, 10 male).

All data was documented in a table. The screenshots of the Instagram posts were also collected. The following analysis parameters were collected: product name, country of manufacture, active ingredients, dosage form, daily therapy costs (DTC), dosages of the active ingredients, vitamin and mineral overdoses based on various reference values (see Tables 1 and 2), dosage information in % of the daily recommended reference amount (Verordnung (EU) Nr. 1169/2011) (post and manufacturer), overdose warnings (post and manufacturer), information on adverse drug reactions, drug interactions and contraindications (post and manufacturer), discount codes, suggestiveness of product names in relation to a false or exaggerated effect, and influencer claims of efficacy. The data was examined using these analysis parameters and then evaluated.

**Table 1:** Recommended maximum daily amounts in dietary supplements, tolerable upper intake levels (UL), and daily reference amounts for vitamins from the BfR report “Aktualisierte Höchstmengenvorschläge für Vitamine und Mineralstoffe in Nahrungsergänzungsmitteln und angereicherten Lebensmitteln,” the “Overview on Tolerable Upper Intake Levels as derived by the Scientific Committee on Food (SCF) and the EFSA Panel on Dietetic Products, Nutrition and Allergies (NDA)” from the EFSA and the Verordnung (EU) Nr. 1169/2011)

Vitamin	Recommended maximum daily amount in dietary supplements (BfR 2021)—15–65 years	UL (EFSA 2018)—adults	Daily reference quantity (BfR 2021)—adults		Daily reference quantity (Verordnung (EU) Nr. 1169/2011)
			Men	Woman	
Vitamin A	200 µg	3 mg	850 µg	700 µg	800 µg
Vitamin C	250 mg	No adequate data	110 mg	95 mg	80 mg
Vitamin D	20 µg	100 µg (4000 I.U.)	15 µg		5 µg
Vitamin E	30 µg	300 mg	15 mg	12 mg	12 mg
Vitamin K	25 µg	No adequate data	70 µg	60 µg	75 µg
Thiamine	No maximum quantities	No adequate data	1.1–1.3 mg	1 mg	1.1 mg
Riboflavin	No maximum quantities	No adequate data	1.3–1.4 mg	1–1.1 mg	1.4 mg
Niacin	160 mg / Pregnant women 16 mg	900 mg	14–16 mg	11–13 mg	16 mg
Pantothenic acid	No maximum quantities	No adequate data	6 mg		6 mg
Vitamin B_6_	3.5 mg	25 mg	1.6 mg	1.4 mg	1.4 mg
Biotin	No maximum quantities	No adequate data	40 µg		50 µg
Folate	200 µg	1 mg	300 µg		200 µg
Vitamin B_12_	25 µg	No adverse effects	4 µg		2.5 µg

**Table 2:** Recommended maximum daily amounts in dietary supplements, tolerable upper intake levels (UL), and daily reference amounts for minerals from the BfR report “Aktualisierte Höchstmengenvorschläge für Vitamine und Mineralstoffe in Nahrungsergänzungsmitteln und angereicherten Lebensmitteln,” the “Overview on Tolerable Upper Intake Levels as derived by the Scientific Committee on Food (SCF) and the EFSA Panel on Dietetic Products, Nutrition and Allergies (NDA)” from the EFSA and the Verordnung (EU) Nr. 1169/2011

Mineral	Recommended maximum daily amount in dietary supplements (BfR 2021)—15–65 years	UL (EFSA 2018)—adults	Recommended daily reference amount (BfR 2021)—adults		Recommended daily reference amount (Verordnung (EU) Nr. 1169/2011)
			Men	Woman	
Boron	0.5 mg	10 mg	11.2 mg		No details yet
Calcium	500 mg	2500 mg	1000 mg		800 mg
Chlorides	No addition!	No adequate data	2300 mg		800 mg
Chromium	60 µg	No adequate data	30–100 µg		40 µg
Copper	1 mg	5 mg	1–1.5 mg		1 mg
Fluorides	No addition!	7 mg	3.8 mg	3.1 mg	3.5 mg
Iodine	100 µg	600 µg	200 µg		150 µg
Iron	6 mg	No adequate data	10 mg	15 mg	14 mg
Magnesium	250 mg	250 mg	350 mg	300 mg	375 mg
Manganese	0.5 mg	No adequate data	2–5 mg		2 mg
Molybdenum	80 µg	600 µg	50–100 µg		50 µg
Phosphorus	No addition!	No adequate data	700 mg		700 mg
Potassium	500 mg	No adequate data	4000 mg		2000 mg
Selenium	45 µg	300 µg	70 µg	60 µg	55 µg
Silicon^13^	See footnote^13^	No adequate data	700 mg		No details yet
Sodium	No addition!	No adequate data	2,000 mg		No details yet
Zinc	6.5 mg	25 mg	14 mg	8 mg	10 mg

^13^Silicon dioxide 350 mg, silicic acid (silica gel) 100 mg, choline-stabilized orthosilicic acid 10 mg, organic silicon (monomethylsilanetriol) 10 mg

**Fig. 2:**
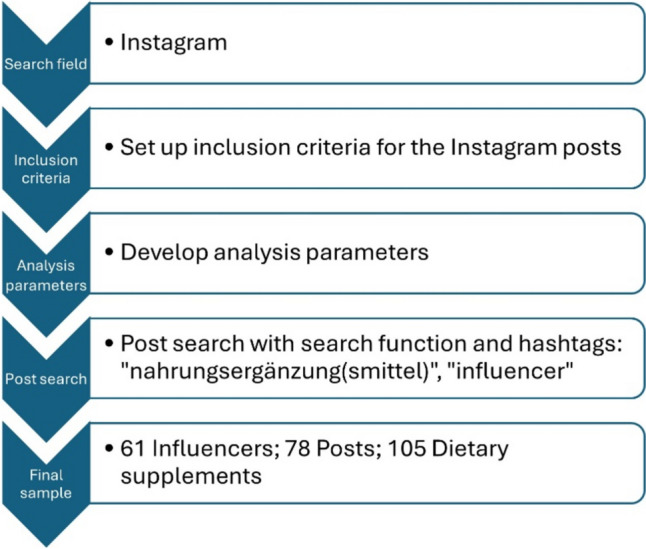
Flow chart of the process for quantitative sample extraction

Figure 2 shows a flow chart of the process for quantitative sample extraction.

## Results and Discussion

### Country of manufacture/country of origin of the preparations

**Fig. 3:**
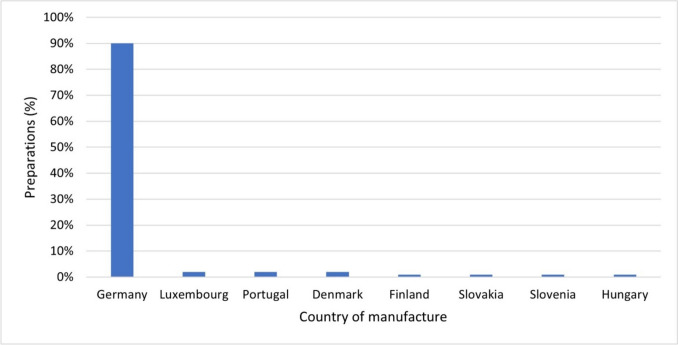
Country of manufacture/country of origin of all preparations as a bar chart

Figure 3 shows the countries of manufacture/country of origin of the preparations analyzed. 90% (95) of the dietary supplements indicated Germany as the country of manufacture/country of origin. The remaining preparations (10 preparations; 10%) were from other EU countries.

All preparations (105) were manufactured in an EU country. Dietary supplements are subject to food law. According to the European Parliament’s Food Information Regulation, the indication of the origin of a product is mandatory if the consumer could otherwise be misled as to the actual country of manufacture or place of origin of the dietary supplement. This obligation applies to dietary supplements if the information on origin is necessary to enable the consumer to make an informed decision when choosing the product (Verordnung (EU) Nr. 1169/2011).

In addition, according to an amendment to the Food Information Regulation, the indication of the origin of the primary ingredient of a dietary supplement has been mandatory since April 1, 2020 if this does not correspond to the country of origin or place of origin of the final product (Durchführungsverordnung (EU) Nr. 2018/775).

A country of manufacture/country of origin was indicated for all the products analyzed. This means that the consumer can obtain precise information about the manufacturing standards and regulations for dietary supplements in the respective country. It is essential for the consumer to know the country from which the dietary supplement originates and to obtain precise information about its manufacture. This enables them to make an informed decision about which dietary supplement to buy.

German influencers predominantly advertised dietary supplements that are produced in Germany. This could indicate that German influencers have a high level of trust in national production standards. At the same time, it is easier for German companies to attract German influencers for advertising partnerships as there are no language barriers.

EU regulations, in particular Verordnung (EU) Nr. 1169/2011 and Durchführungsverordnung (EU) Nr. 2018/775), play a central role in transparency and consumer protection. The findings emphasize the importance of clear and uniform regulations within the EU to ensure a level playing field. This is particularly important for the free movement of goods and ensuring high standards throughout the EU.

### Active ingredients

A total of 857 active ingredients were found in 105 analyzed dietary supplements, including 238 different active ingredients. 23% of the preparations analyzed (24) contained only one active ingredient and were therefore mono-preparations. 77% of the preparations (81) contained more than one active ingredient and were combination preparations. On average, each preparation contained 8.16 active ingredients.

**Fig. 4:**
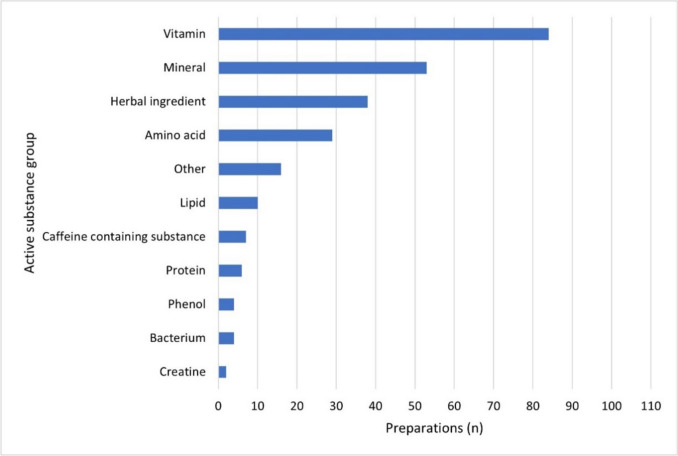
Distribution of the main active substance groups in the preparations as a horizontal bar chart

Figure 4 shows the eleven main groups into which the 238 different active substances were divided. For example, 84 dietary supplements (80%) contain at least one vitamin.

The preparations most frequently contained an active ingredient from the main group ‘Vitamins’ (84 preparations; 80%). This was followed by the main group ‘Minerals’ (53 preparations; 50%). In a survey conducted by Statista in 2022, these two main groups were also the most popular among the Germans surveyed (Statista Daily Data, Brandt 2023).

**Fig. 5:**
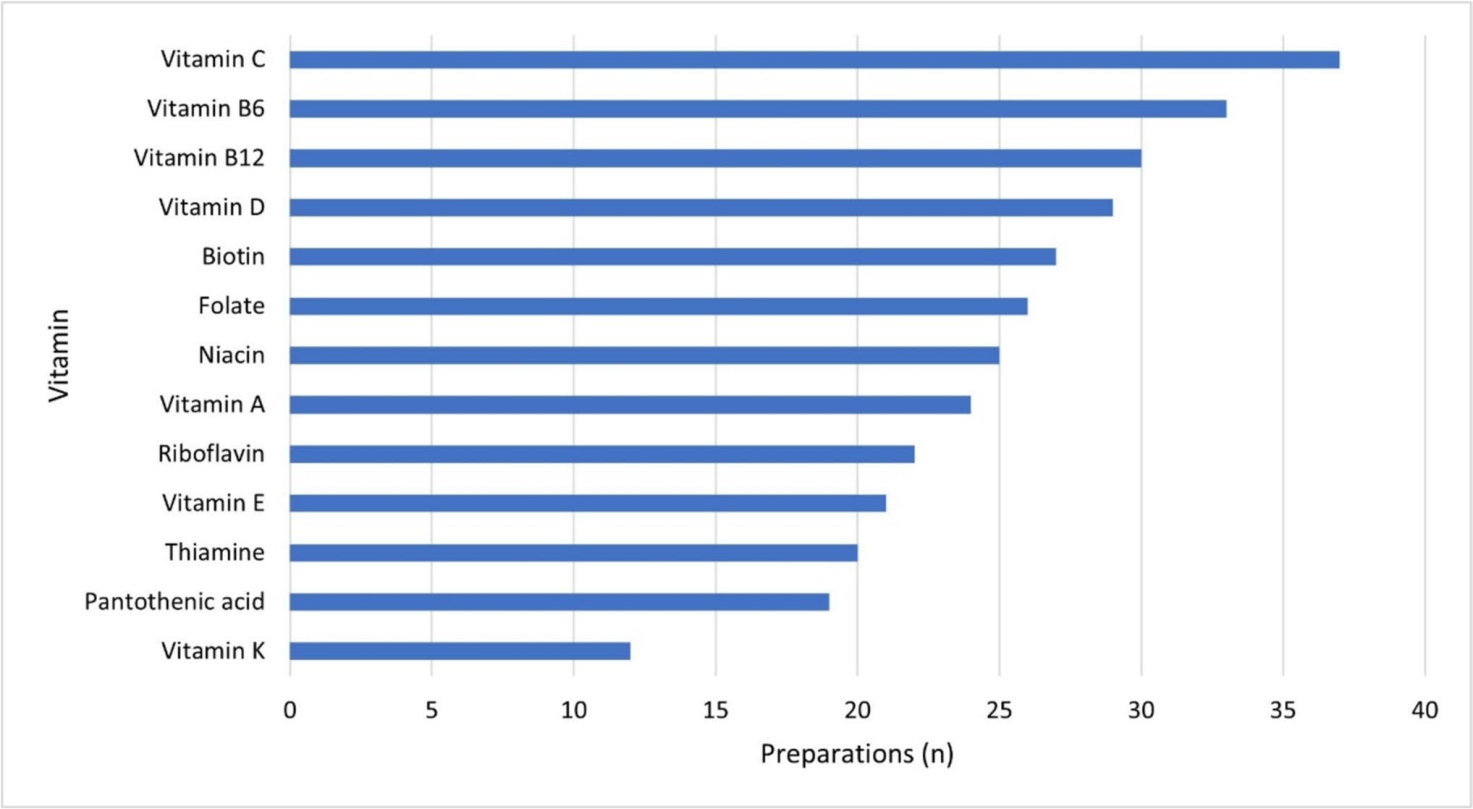
Distribution of the specific vitamins within the main substance group ‘Vitamins’ based on the number of preparations containing the respective vitamin, as a horizontal bar chart

Figure 5 shows the individual components of the main group ‘Vitamins’ divided into their specific 13 vitamins in detail.

Vitamin C, vitamin B_6_, and vitamin B_12_ were the three most frequently found vitamins in this study. Closely followed by vitamin D. This result is largely in line with the findings from the BfR consumer monitor from 2021, where the Germans surveyed stated that they mainly supplement vitamin D (45% of respondents), vitamin B_12_ (36% of respondents), and vitamin C (32% of respondents). Multivitamin supplements were also popular, taken by 28% of respondents (BfR 2021).

**Fig. 6:**
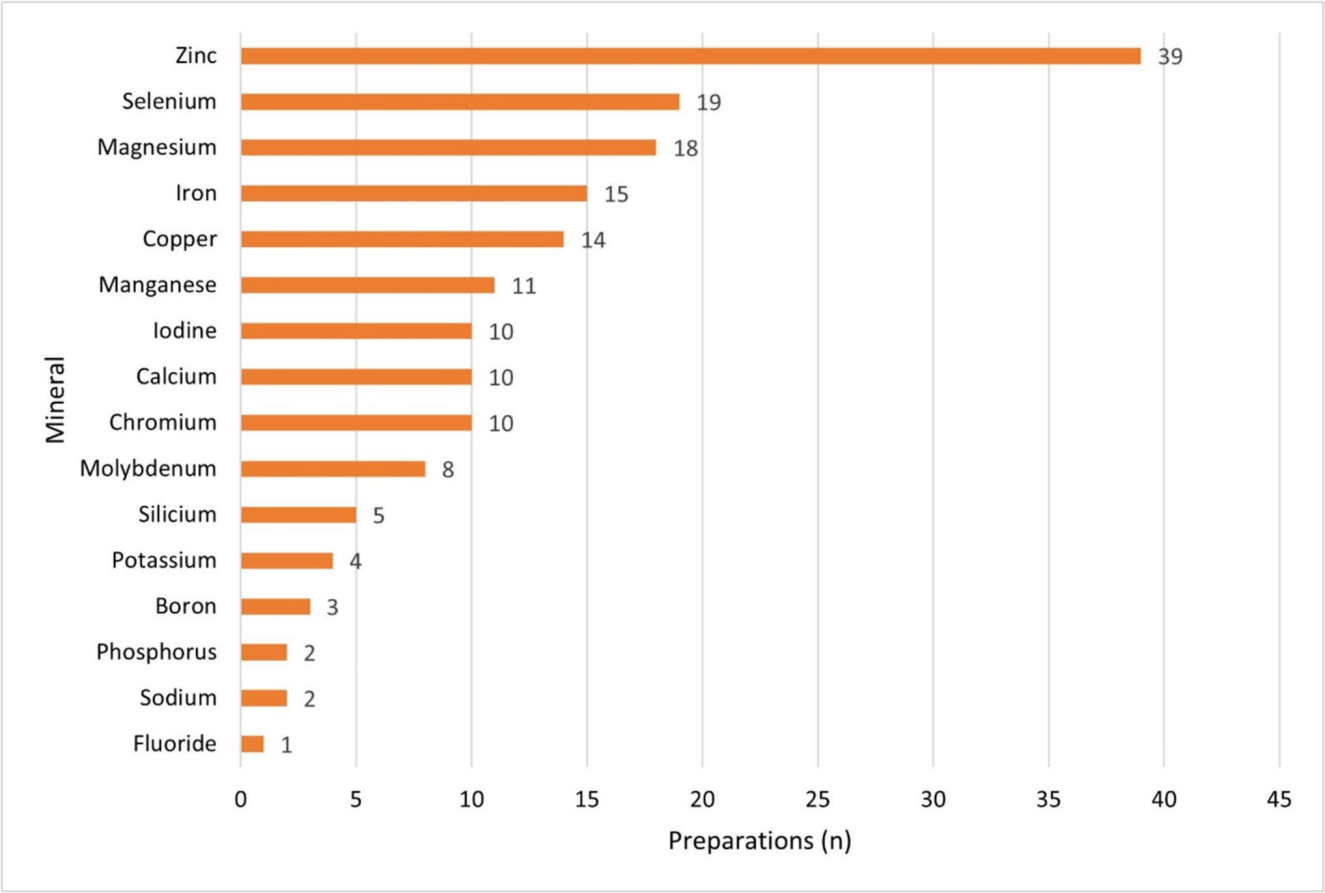
Breakdown of the specific minerals within the main substance group ‘Minerals’ based on the number of preparations containing the respective mineral as a horizontal bar chart

Figure 6 shows the individual components of the main group “Minerals” subdivided into their specific 16 minerals.

Zinc (39 preparations; 37%) was the most frequently used active ingredient among all (238) different active ingredients.

Overall, there was a wide variety of ingredients contained in the preparations. This allows manufacturers to attend to the different needs and preferences of consumers. At the same time, preparations containing several ingredients—combination preparations—dominated. On average, each preparation contained 8.16 different active ingredients—a worrying amount. Manufacturers offer consumers the opportunity to take various ingredients in one preparation. However, these dietary supplements should be consumed with caution. If no nutrient deficiency is detected and supplements are taken regularly, individual active ingredients can be overdosed or interact with medications, for example. In addition, taking several combination dietary supplements containing the same active ingredients can unknowingly lead to an overdose of the active ingredients. Consumers should inform themselves about the possible risks, especially before taking preparations that contain many different active ingredients. In addition, it could be useful to limit the number of active ingredients contained in dietary supplements to avoid undesirable effects and interactions between the individual active ingredients. To this end, influencers advertising the products should point out that dietary supplements should only be taken after careful clarification.

### Dosage form of the preparations

**Fig. 7:**
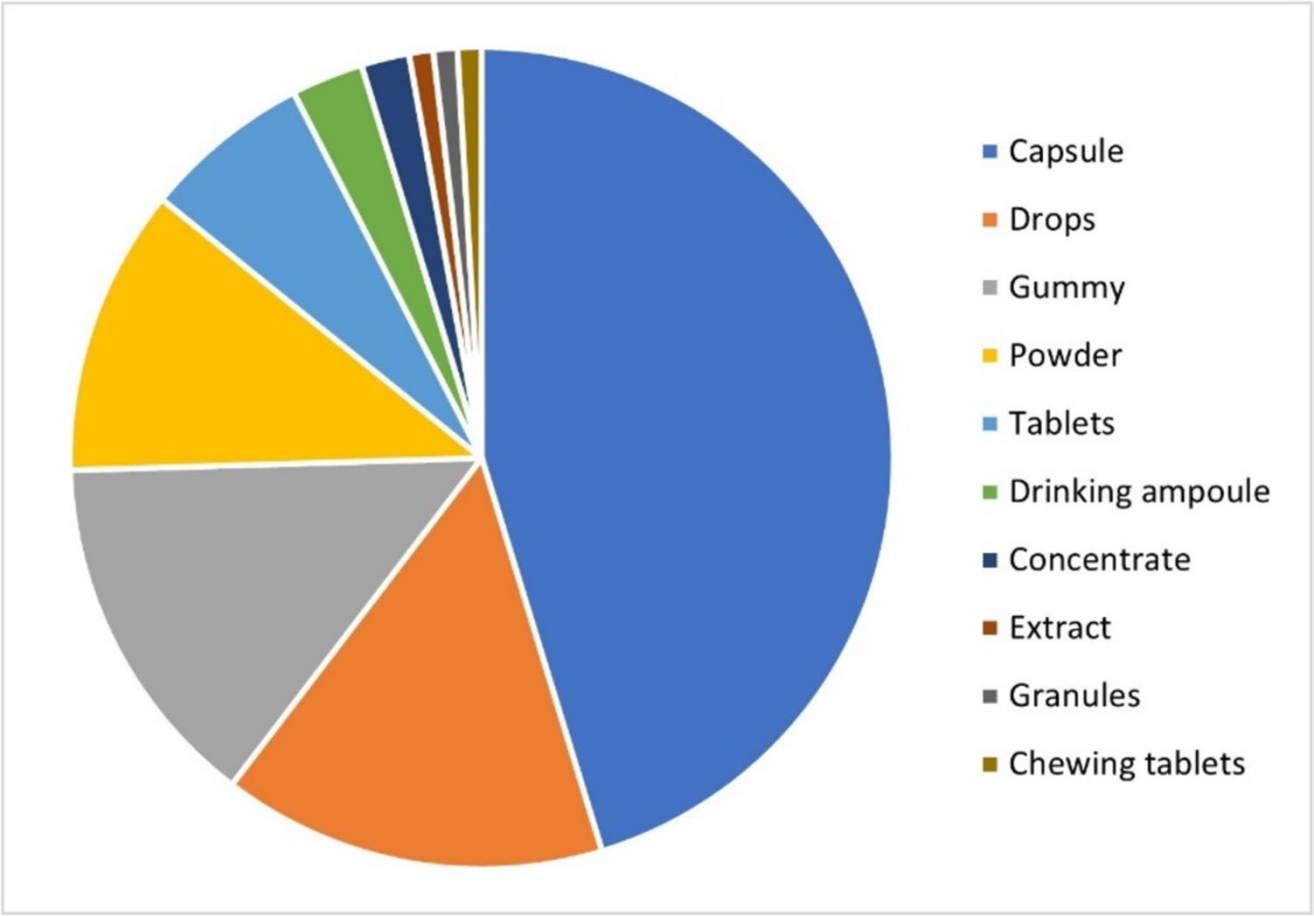
Distribution of the dosage forms of the preparations as a pie chart

Figure 7 shows the distribution of the dosage forms of the preparations.

Capsules were the most popular dosage form. They are easy to take, can be dosed precisely, and have good stability. However, they are not suitable for people with swallowing difficulties and the onset of action is slower than with drops. Drops are also suitable for people with swallowing difficulties. Although dietary supplements in the form of drops can be dosed variably, there is a risk of overdosing as the recommended number of drops can easily be exceeded or counting errors can occur. Wine gums offer a tasty and more convenient way of supplementing, making them particularly attractive to children or people who have difficulty taking capsules or tablets. However, they can be dangerous to take, especially for children. The wine gums can easily be mistaken for conventional sweets and, if not stored properly, can be consumed in excessive quantities by children. This also carries the risk of overdosing.

In total, the preparations were offered in ten different dosage forms. The wide variety of dosage forms enables manufacturers to meet different consumer preferences and application needs.

### Daily therapy costs (DTC)

**Fig. 8:**
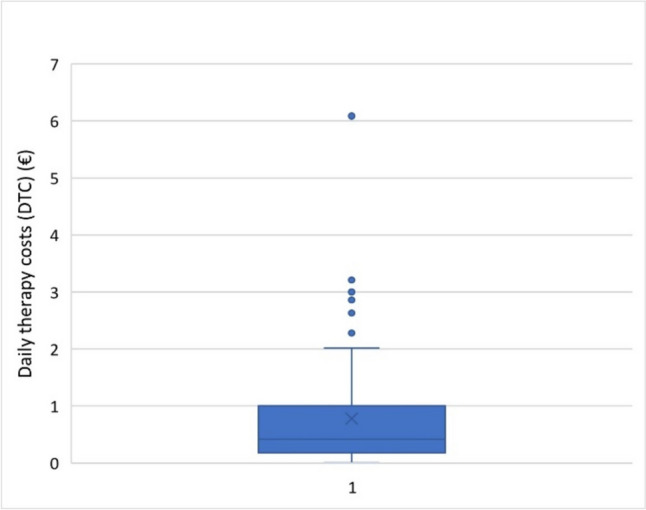
Representation of the daily therapy costs (DTC) (€) of all preparations as a box chart (all prices without discount codes). The box corresponds to the middle 50% of the data (interquartile range). It is delimited by the upper (Q3) and lower quartiles (Q1). The lines in the box indicate the median. The crosses mark the mean value. The upper and the lower whisker extend up to 1.5 times the interquartile range (IQR) above the third quartile (Q3) and below the first quartile (Q1). The dots outside the boxes highlight the outliers

Figure 8 shows the daily therapy costs (DTC) for the analyzed dietary supplements as a box plot (all prices without the use of discount codes).

The average DTC was € 0.78. The median was € 0.42. The DTC for the cheapest preparation was € 0.002, while the DTC for the most expensive preparation was € 6.09.

In addition to the majority of preparations for which the DTC was ≤ € 1 (79 preparations; 75%), there were eight outliers that were above the upper whisker. For these preparations, the DTC were, in ascending order: € 2.28 (1 preparation; 1%), € 2.63 (1 preparation; 1%), € 2.66 (2 preparations; 2%), € 2.86 (1 preparation; 1%), € 3.00 (1 preparation; 1%), € 3.21 (1 preparation; 1%), and € 6.09 (1 preparation; 1%). DTC of € 6.09 result in monthly costs of around € 180, which represents a considerable cost factor.

The wide price range between the cheapest preparation (€ 0.002 DTC) and the most expensive preparation (€ 6.09 DTC) is explained by the fact that dietary supplements are foodstuffs and are therefore not subject to the German Arzneimittelpreisverordnung which regulates pricing (AMPreisV 2023).

The lack of price regulation has resulted in remarkable price variability between the various products. The consequence of this lack of regulation is double-edged: on the one hand, many dietary supplements can be purchased at affordable prices; on the other hand, some dietary supplements are associated with high costs, which can represent a considerable financial burden, especially for people who take dietary supplements on a long-term basis.

Another critical problem is the lack of clarity as to whether the higher costs of the more expensive dietary supplements are in proportion to their benefits. Arbitrary pricing by manufacturers leads to a lack of transparency as to why dietary supplements cost as much as they do.

Although the advertised dietary supplements were shown in every post, the price of only one (1%) of the 105 advertised dietary supplements was mentioned in the post by the influencer. No price information was provided for the remaining 104 (99%) dietary supplements. This lack of transparency makes it difficult for users to make an informed decision about whether they can afford the advertised product. It appears that this practice is aimed at drawing focus to other product features or emphasizing the perceived necessity of the product without deterring potential buyers by disclosing high costs.

There should be improved consumer education and possibly regulatory intervention to ensure fair pricing and transparency in the dietary supplement market. Such measures could help to reduce the price spread and ensure that consumers can make an informed decision based on a clear understanding of the costs and benefits of the products.

### Vitamins - exceeding the reference values

The main group “Vitamins” was the largest main group in the study. At least one vitamin was contained in 80% (84) of all preparations analyzed (105).

As dietary supplements are foods and not medicinal products, there are no prescribed maximum quantities that must be adhered to (BVL). Instead, there are reference values from various institutions.

Table 1 provides an overview of the recommended maximum daily amounts in dietary supplements, tolerable upper intake levels (UL), and daily reference amounts for vitamins.

**Fig. 9:**
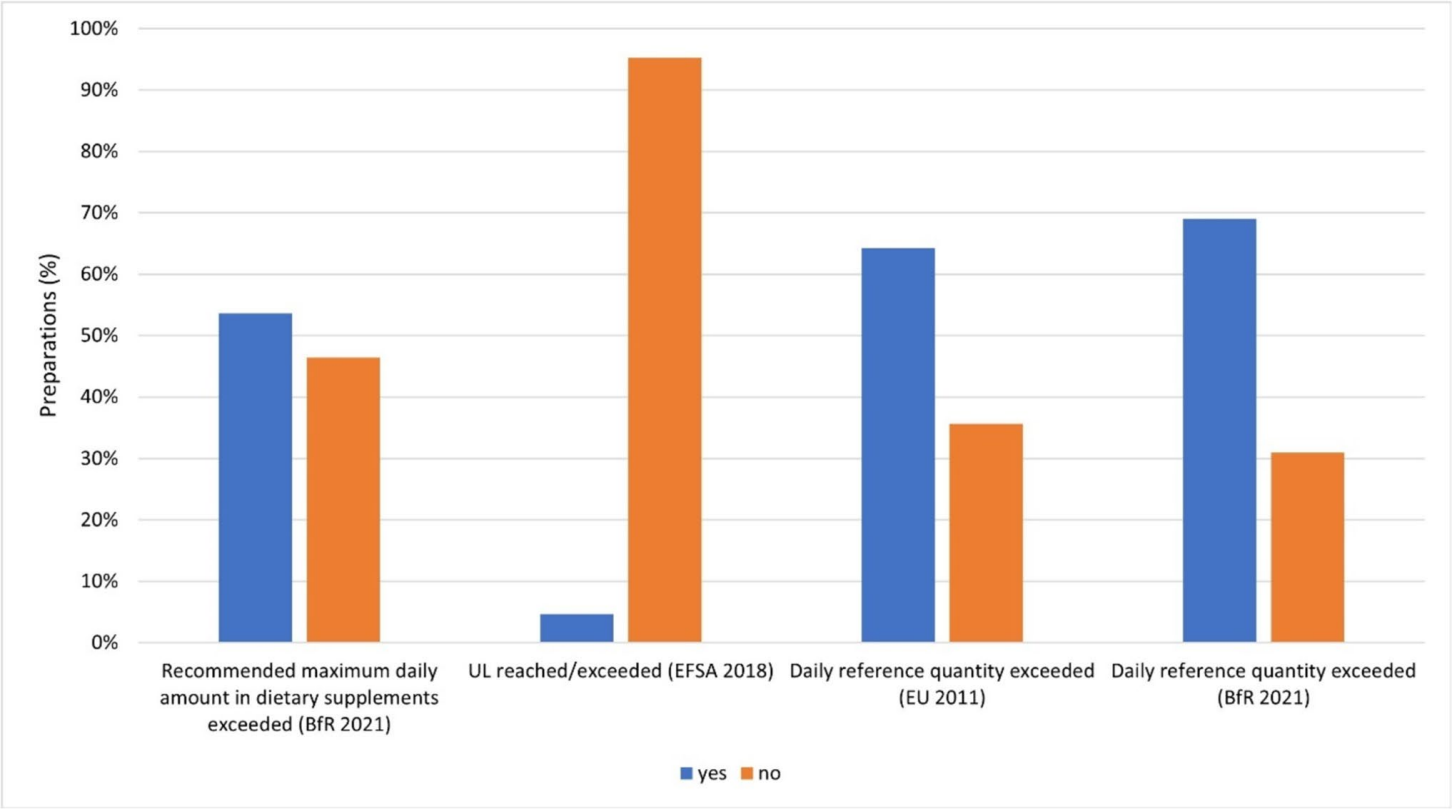
Representation of the frequency of overdoses of vitamin supplements above the recommended maximum daily amounts in dietary supplements, tolerable upper intake levels (UL), and daily reference amounts for vitamins from the BfR, EFSA and Verordnung (EU) Nr. 1169/2011) as a bar chart. The proportion of preparations that exceed the respective reference value is colored blue. The proportion of preparations not exceeding the respective reference value is colored orange

Figure 9 shows how often the doses of the individual vitamins from the vitamin supplements (84 preparations; 80%) exceeded the reference quantity recommendations for vitamins from Table 1.

Forty-five vitamin supplements (54%) exceeded the BfR’s recommended maximum daily amount in dietary supplements with at least one vitamin (BfR 2021). Less than half of the vitamin supplements (39 preparations; 46%) adhered to this limit. Four vitamin supplements (5%) reached or exceeded the UL. The tolerable upper intake level (UL) is the maximum level of total chronic intake of a nutrient from all sources that is unlikely to pose a risk to human health. If the UL is exceeded, potentially toxic effects may result from the corresponding active substance (EFSA NDA Panel, 2022). If the UL is reached by the dosage of a vitamin in a dietary supplement, the intake of any further small proportion of this vitamin leads to the UL being exceeded and to potentially toxic effects. Most vitamin supplements did not meet or exceed the UL (80 preparations; 95). The recommended daily reference intakes from the Verordnung (EU) Nr. 1169/2011) were exceeded by 54 vitamin supplements (64%) containing at least one vitamin. The latest recommendation for daily reference intakes comes from the BfR from 2021 (BfR 2021). Fifty-eight vitamin preparations (69%) exceeded this recommended daily reference amount of the BfR for the respective vitamin with at least one vitamin.

### Minerals - exceeding the reference values

The main group “Minerals” was the second largest main group in the study. Half (53 preparations; 50%) of all preparations analyzed (105) contained at least one mineral substance. A total of 171 minerals were contained in all preparations, an average of 1.6 minerals per preparation. There are also recommended reference values for minerals.

Table 2 provides an overview of the recommended maximum daily amounts in dietary supplements, tolerable intake levels (UL), and daily reference amounts for minerals.

**Fig. 10:**
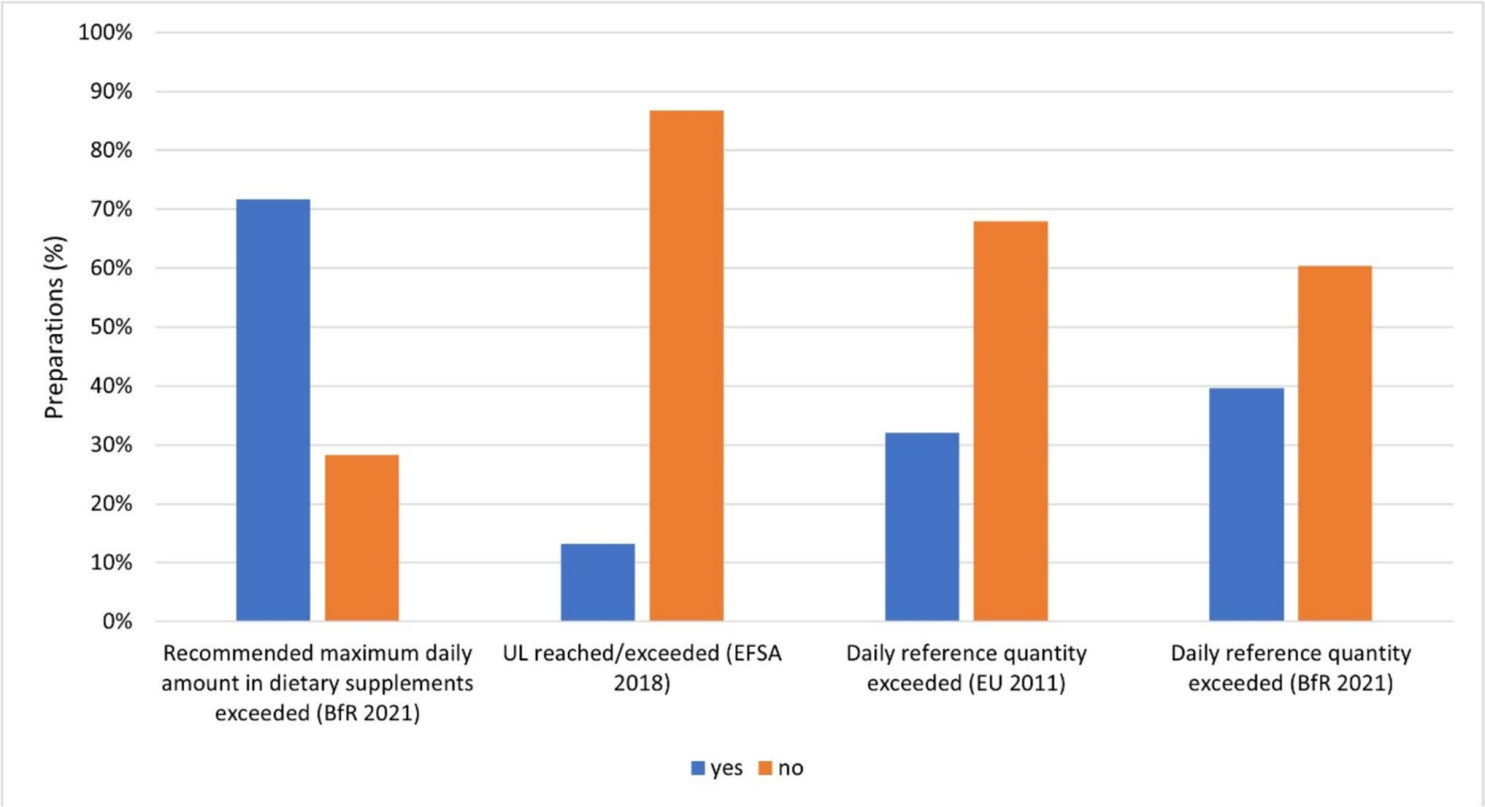
Representation of the frequency of overdoses of mineral supplements above the recommended maximum daily amounts in dietary supplements, tolerable upper intake levels (UL), and daily reference amounts for vitamins from the BfR, EFSA and Verordnung (EU) Nr. 1169/2011 as a bar chart. The proportion of preparations that exceed the respective reference value is colored blue. The proportion of preparations not exceeding the respective reference value is colored orange

Figure 10 shows how often the dosages of the individual minerals from the mineral supplements (53 preparations; 50%) exceeded the recommended amounts for minerals from Table 2.

Thirty-eight mineral supplements (72%) exceeded the maximum daily amount recommended by the BfR for dietary supplements with at least one mineral (BfR 2021). Not even a third of the mineral supplements (15 preparations; 28%) adhered to this limit. Seven mineral supplements (13%) reached or exceeded the UL, which can lead to toxic effects from the corresponding active ingredient (EFSA 2018). The majority of mineral supplements did not reach or exceed the UL (46 preparations; 87%). The recommended daily reference intake of the Verordnung (EU) Nr. 1169/2011 was exceeded by 17 mineral supplements (32%) with at least one mineral. 21 mineral preparations (40%) exceeded the BfR’s recommended daily reference amount for the respective mineral with at least one mineral (BfR 2021).

Every reference value was exceeded by at least one vitamin and mineral in the analyzed dietary supplements. More than half of the vitamin supplements (45 preparations; 54%) and most of the mineral supplements (38 preparations; 72%) contained doses that exceeded the amounts considered safe in the BfR report “Aktualisierte Höchstmengenvorschläge für Vitamine und Mineralstoffe in Nahrungsergänzungsmitteln und angereicherten Lebensmitteln.” Reaching or exceeding the UL is particularly problematic, as chronic intake of such amounts can have potentially serious health consequences.

Furthermore, if consumers do not adhere to the manufacturer’s recommended intake and take more than the recommended daily portion of a dietary supplement, the risk of overdosing on individual vitamins and minerals increases, which can lead to potentially toxic effects. These findings underline the need to strictly monitor the dosage of vitamins in dietary supplements and, if necessary, to take regulatory measures to improve consumer protection.

Although the labeling requirements under the NemV and the Verordnung (EU) Nr. 1169/2011 provide some guidance, consumer safety is not sufficiently guaranteed if the actual dosages are in many cases far above the recommended values. In addition, the labeling requirements refer to the reference values from Verordnung (EU) Nr. 1169/2011 from 2011. However, current reference values for vitamins and minerals, as published by the BfR in 2021 are already available (BfR 2021). It would therefore make sense to introduce binding maximum levels for vitamins and minerals in dietary supplements based on the latest scientific findings in order to minimize the risk of overdoses and the associated health risks. In addition, increased consumer education—especially through influencers on platforms such as Instagram—about the potential risks of excessive vitamin and mineral intake could lead to a more conscious approach to dietary supplements.

### Vitamins and minerals - UL reached/exceeded/overdosing

Table 3 shows the individual vitamins and minerals of the analyzed preparations that reached and exceeded the current tolerable upper intake level (UL) of the EFSA 2018 from the report “Overview on Tolerable Upper Intake Levels as derived by the Scientific Committee on Food (SCF) and the EFSA Panel on Dietetic Products, Nutrition and Allergies (NDA).”

**Table 3:** Overview of vitamins and minerals with associated UL, their dosage, and reaching or exceeding the UL (EFSA 2018). Exceeding the UL in red digits

Active ingredient	UL (EFSA 2018)	Dosage—preparation	UL exceeded/achieved
Vitamin A	3000 µg	3620 µg	620 µg = 20.6%
Vitamin D	100 µg (4000 I.U.)	125 µg (5000 I.U.)	25 µg = 25%
Vitamin B_6_	25 mg	1) 25mg	achieved
		2) 25 mg	achieved
Magnesium	250 mg	1) 402 mg	152 mg = 60.8%
		2) 400 mg	150 mg = 60%
		3) 375 mg	125 mg = 50%
		4) 375 mg	125 mg = 50%
		5) 375 mg	125 mg = 50%
Zinc	25 mg	1) 25 mg	Achieved
		2) 25 mg	Achieved

The UL is the maximum level for the total chronic intake of a nutrient from all sources that is unlikely to pose a risk to human health. If the value is exceeded, potentially toxic effects may arise from the corresponding active substance (EFSA NDA Panel, 2022).

The UL for vitamin A is 3000 µg/day (10,000 I.U.) (EFSA 2018). Of 24 preparations containing vitamin A, one preparation exceeded the UL with a dose of 3620 µg (approx. 12,000 I.U.). The UL was thus exceeded by 21% (620 µg/2000 I.U.). In addition to hepatotoxicity (Biesalski 1989; EFSA 2006; Hathcock et al. 1990; Nollevaux et al. 2006), teratogenicity (EFSA 2006; Hayes et al. 1981; Hendrickx et al. 2000; Rothman et al. 1995) has also been described for chronic intake of high-dose vitamin A (≥ 3000 µg/day). With a chronic intake of ≥ 10,000 I.U./day, visual impairment and severe intracranial bleeding have also been described (Moses 2021). In pregnancy, Moses describes that chronic intake of ≥ 10,000 I.U. vitamin A per day is associated with an increased incidence of cranial malformations and abnormalities of the central nervous system, heart, and limbs (Moses 2021). Furthermore, vitamin A intake of ≥ 3000 µg/day during pregnancy is associated with a 4.8-fold increase in the risk of neural tube defects (Rothman et al. 1995).

The UL for vitamin D is 100 µg (4000 I.U.)/day (EFSA 2018). One of the 29 preparations containing vitamin D had a dosage of 125 µg and thus exceeded the UL for vitamin D by 25 µg/1000 I.U. (25%). Toxicity due to excessive vitamin D intake can result from hypercalcemia. Acute toxicity can occur at vitamin D doses exceeding 10000 IU per day, leading to serum 25-hydroxyvitamin D concentrations above 150 ng/ml (>375 nmol/l). This can lead to vomiting, constipation and polyuria, among other things (Marcinowska-Suchowierska et al. 2018). Chronic vitamin D intake of more than 4000 I.U./day (over many years) has been reported to cause kidney stones and mineral deposits in the tissue (Janoušek et al. 2022; Rizzoli 2021). Reduced bone density has also been described with a chronic vitamin D intake of above 4000 I.U./day (Moses 2021; Pilz et al. 2018; Rizzoli 2021) and even a higher risk of falls, especially for the elderly (Pilz et al. 2018; Rizzoli 2021). There is no clear threshold above which hypercalcemia occurs. Serum 25-hydroxyvitamin D concentrations from 375 nmol/l are frequently cited for the occurrence of hypercalcemia (Janoušek et al. 2022; Marcinowska-Suchowierska et al. 2018; Pilz et al. 2018).

The UL for vitamin B6 is 25 mg/day (EFSA 2018). Two of the 33 preparations containing vitamin B6 each reached the UL with a dosage of 25 mg. Any further small intake of vitamin B6 leads to the UL being exceeded and thus to potentially toxic effects. Neurotoxicity, which manifests as peripheral polyneuropathy, is described with chronic vitamin B6 intake for doses of ≥ 200 mg/day (Moses 2021) or ≥ 500 mg/day (EFSA 2006). If the two preparations analyzed are taken as prescribed and not abused in excessive quantities, the doses are far from the threshold for harmful effects.

The UL for magnesium is 250 mg/day (EFSA 2018). Five of the 18 preparations containing magnesium exceeded the UL. In descending order, the UL was exceeded by 152 mg (61%), 150 mg (60%), and 3x 125 mg (50%). Symptoms of hypermagnesemia such as hypotension, nausea, vomiting, stomach cramps, flushing, urinary retention, ileus, depression, and lethargy are possible with serum concentrations of 1.74–2.61 mmol/l from high-dose dietary supplements (Moses 2021).

The UL for zinc is 25 mg/day (EFSA 2018). Two of the 39 preparations containing zinc reached the UL with a dosage of 25 mg each. The EFSA describes vegetative symptoms such as nausea, vomiting, upper abdominal pain, stomach cramps, and diarrhea with high-dose zinc supplementation (EFSA 2006). These symptoms are also described by Moses with chronic zinc supplementation of ≥ 40 mg/day (Moses 2021). In children, doses of ≤ 20 mg/day can already lead to vomiting (Li et al. 2022). In a mouse model in which the mice were fed a high-dose zinc solution of 60 ppm (60 mg zinc/l), zinc supplementation led to a selective zinc deficiency in the hippocampus, which impaired the mice’s learning and memory performance (Yang et al. 2013).

A total of seven dietary supplements (7%) exceeded the Upper Intake Level (UL), and a further four supplements (4%) reached this critical value. Chronic intake of high-dose dietary supplements can entail considerable health risks. For this reason, dietary supplements should only be taken after careful consideration of the costs and benefits, for example in the case of a diagnosed vitamin or mineral deficiency that cannot be remedied by a change in diet.

From a consumer protection perspective, strict regulation of dosages in dietary supplements that prohibits exceeding the UL were reasonable. Setting binding maximum levels for vitamins and minerals in dietary supplements could minimize the risk of toxic effects due to overdoses and thus significantly improve consumer protection.

If such maximum levels are not set, it would be even more important that manufacturers attach appropriate warnings if the UL is exceeded or do not place the dietary supplement in question on the market in the first place. This responsibility clearly lies with the manufacturers.

In addition, it is essential to educate consumers about the importance of the UL and the potential toxic effects of exceeding it. Influencers could play a key role in this by using their reach on social media to help spread this important health information.

### User-friendliness - specification of the preparation dose (%) of the daily dose recommended by Verordnung (EU) Nr. 1169/2011

**Fig. 11:**
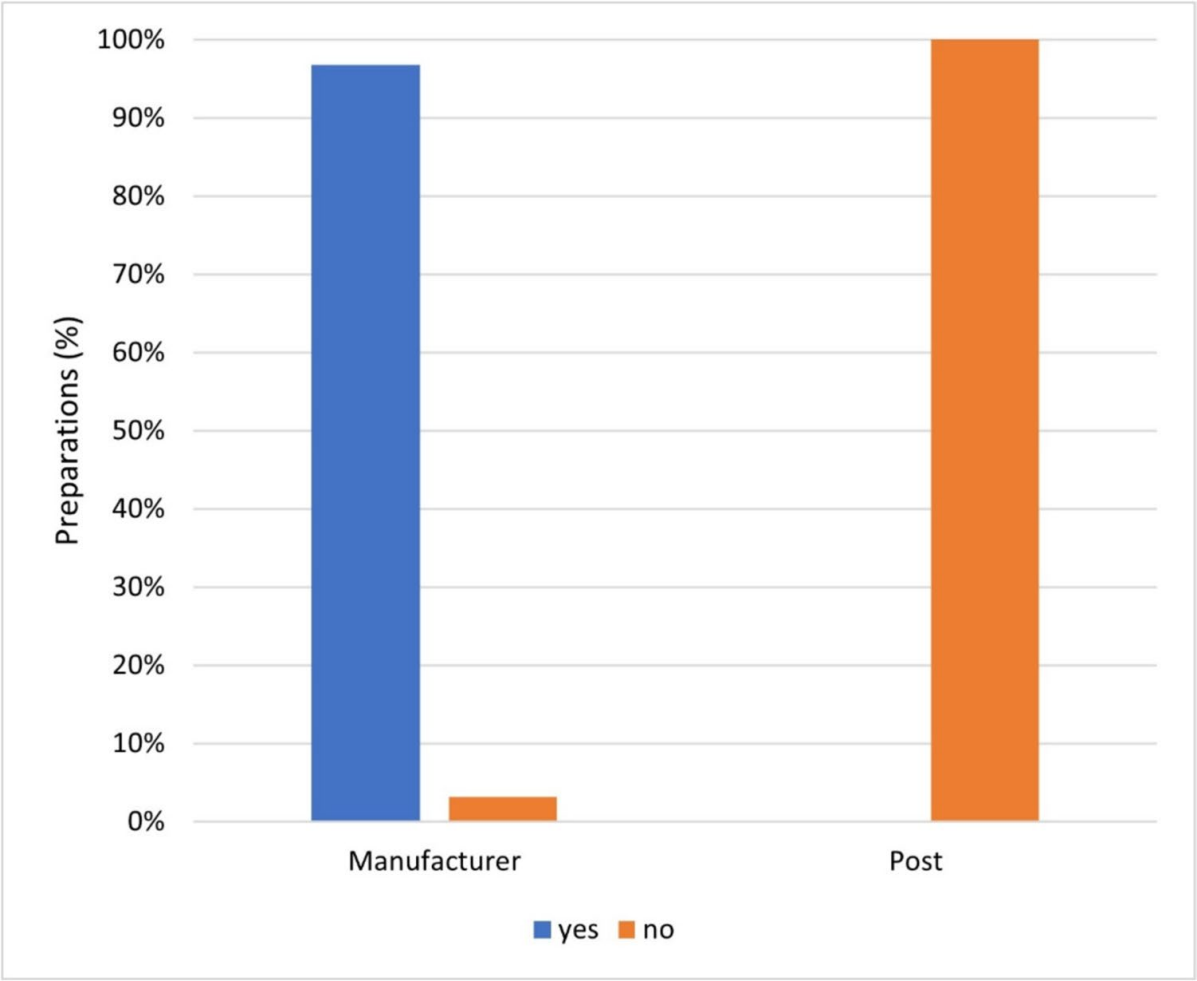
Representation of the frequency of user-friendliness of the dosage information of the preparations by the manufacturer and in the posts as a bar chart, whereby the proportion of preparations with user-friendly dosage information is colored blue, and the proportion of preparations without is colored orange

Figure 11 shows how often manufacturers and influencers provided user-friendly information on the dose of the dietary supplement advertised.

Manufacturers are obliged to provide dosage information as a percentage of the reference values of the respective vitamin and mineral specified in the Verordnung (EU) Nr. 1169/2011 (NemV, §4 (3)). 97% of the preparations provided dosage information. Only 3% of the advertised preparations did not contain the dosage information. In contrast, not a single influencer provided information about the dosage of the advertised preparation in the post. The consumer receives no information on Instagram as to whether it is a high or low-concentration dietary supplement.

Without the dosage information as a percentage of the recommended reference value (Verordnung (EU) Nr. 1169/2011), it is difficult for consumers to assess whether they can cover their needs with the advertised preparation or whether they are even excessively overdosing. Since 54 vitamin supplements (64%) and 17 mineral supplements (32%) exceeded this reference value, it is irresponsible for influencers not to provide any information on the dosage of the dietary supplements. Influencers are the first point of contact with dietary supplements for many people. They have a certain responsibility towards consumers and should therefore provide important information about the dietary supplements. Mandatory information on the dosage of advertised products on Instagram should be considered. The dosage information should refer to official reference values—such as those of the Verordnung (EU) Nr. 1169/2011. To verify the dosage of ingredients in the dietary supplements advertised, consumers should therefore visit the manufacturer’s website, where mandatory dosage information can almost invariably be found.

### Overdose warning for the preparations

**Fig. 12:**
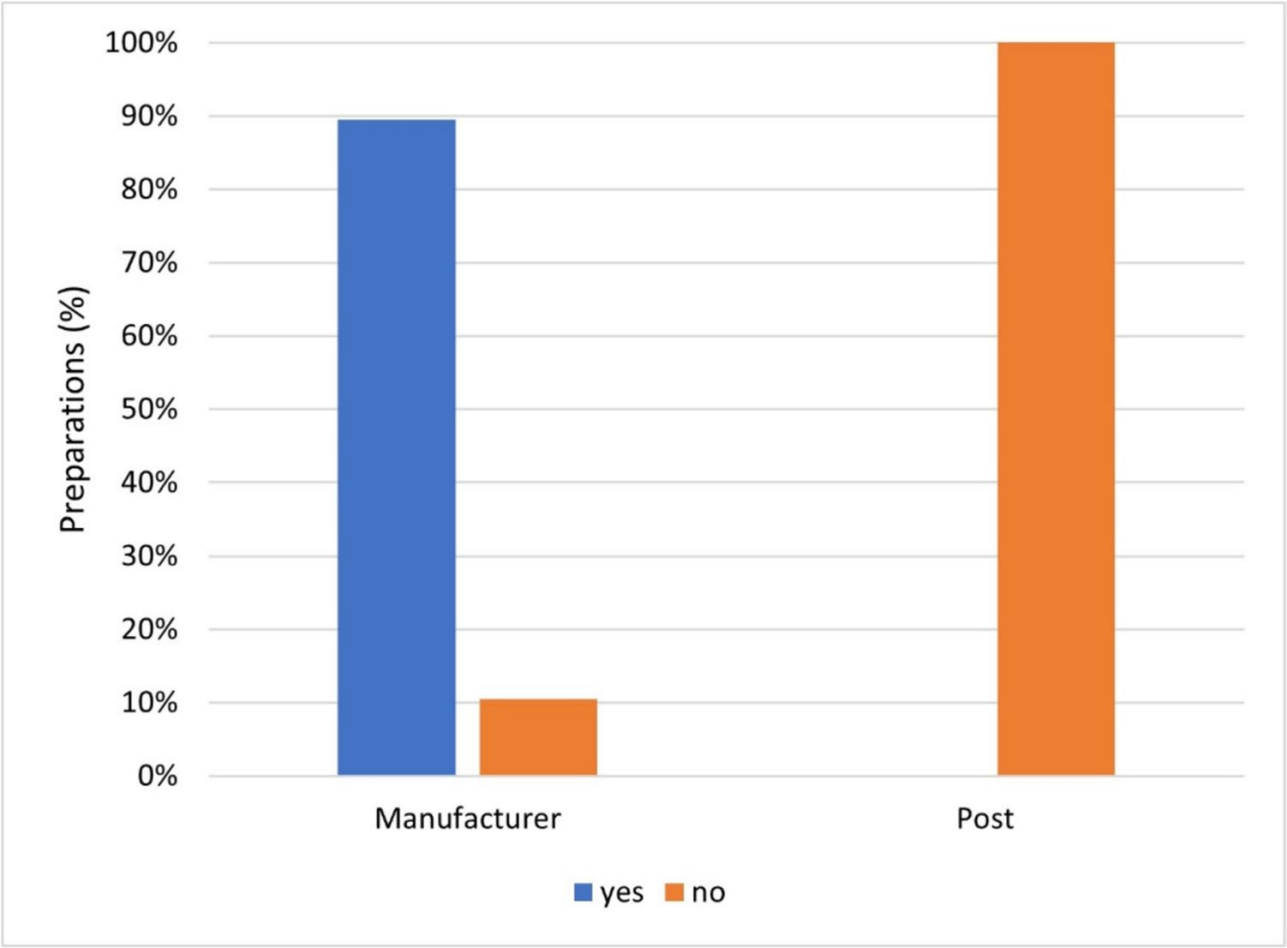
Representation of the frequency of an overdose warning of the preparations by the manufacturer and in the posts as a bar chart, whereby the proportion of preparations with an overdose warning is colored blue, and the proportion of preparations without is colored orange

Figure 12 shows how often manufacturers and influencers warned of an overdose from the advertised dietary supplement.

Manufacturers are obliged to affix the warning “The recommended daily intake must not be exceeded” (NemV, §4 (2)).

In 94 preparations (90%), the manufacturers included an overdose warning. Eleven preparations (10%) did not contain this warning. In contrast, no influencer provided an overdose warning. The fact that the dietary supplements advertised exceed various reference values for vitamins and minerals is illustrated in Figs. 9 and 10. Among the people surveyed by the BfR in 2021, 78% of respondents rated the health risk of vitamins as dietary supplements as “medium” or “(very) low.” At the same time, 70% of respondents felt “moderately” or “(not at all) well” informed about the health risk of vitamins as dietary supplements (BfR 2021).

If influencers do not point out that dietary supplements can be overdosed, it can give consumers the impression that these products do not pose any risks. Since dietary supplements are available over-the-counter, many consumers underestimate the potential health risk of overconsumption. Influencers have a responsibility to educate their followers about the possibility of overdose to avoid harm to health. By not providing transparent information, influencers are accepting health risks for consumers. Consideration should be given to introducing mandatory labeling with overdose warnings for advertised products on Instagram to ensure consumer protection.

### Information on adverse effects, drug interactions, and contraindications

**Fig. 13:**
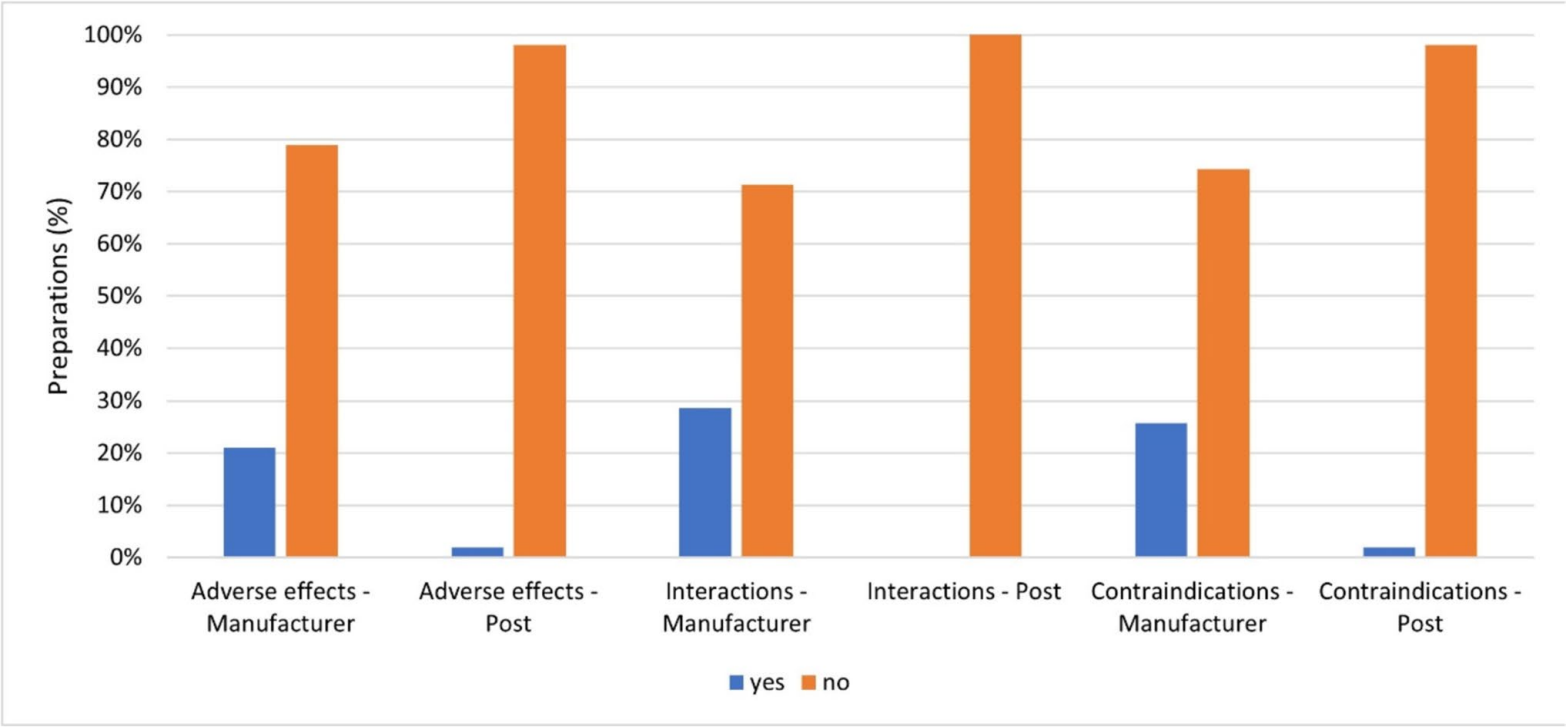
Representation of the frequency of information on adverse effects, drug interactions, and contraindications from the manufacturer and in the posts as a bar chart, whereby the proportion of preparations with an adequate information is colored blue and the proportion of preparations without is colored orange

Figure 13 provides an overview of how often manufacturers and influencers provided information on adverse effects, drug interactions and contraindications of the advertised preparations in the posts. This information is not mandatory.

The manufacturers only stated possible adverse effects for 22 preparations (21%). Most of the preparations (83 preparations; 79%) did not contain this information. In the posts, the influencers only mentioned possible adverse effects for two preparations (2%). 103 preparations (98%) were advertised without any information on possible adverse effects. For just under a third of the preparations (30 preparations; 29%), the manufacturers provided information on drug interactions. 75 preparations (71%) contained no information on drug interactions. The influencers’ posts did not even mention possible drug interactions at all. The same result was shown for the information on contraindications. The manufacturers provided information on contraindications for 27 preparations (26%). Most of the preparations (78 preparations; 74%) were advertised by the manufacturers without any information on contraindications. The influencers provided information on contraindications for only 2 preparations (2%). 103 preparations (98%) were advertised without any information on possible contraindications.

Overall, the manufacturers provided significantly more information on adverse effects, drug interactions and contraindications than in the influencers’ posts. Nevertheless, the manufacturers drew significantly less attention to this than to the overdose warning and the dosage information, both of which are mandatory. Information on possible adverse effects, drug interactions, and contraindications is potentially more of a deterrent for consumers. This could be the reason why manufacturers draw attention to these points much less frequently, even though they represent important health information for the consumer. The influencers consistently provided almost no information on adverse effects, drug interactions, and contraindications—just as little as on dosage information and overdose warnings. In Pilgrim and Bohnet-Joschko’s analysis of Instagram posts from the 50 most popular German fitness influencers, they also came to the conclusion that the products presented are commented on or questioned to a relatively small extent. The focus was primarily on the person and personality of the influencer (Pilgrim and Bohnet-Joschko 2019). Important health information about the dietary supplement, such as adverse effects, drug interactions and contraindications, was neglected. In a retrospective study examining the most viewed YouTube videos on multivitamin supplements, over 80% of videos (80.4%, 95% CI 72.5%, 88.3%) emphasized the benefits of supplements and 72.2% (95% CI 63.3%, 81.1%) recommended viewers to take multivitamin supplements. Over 84% (84.5%, 95% CI 77.3%, 91.7%) of the videos did not mention any risks associated with taking a particular vitamin or dietary supplement (Basch et al. 2016). These two studies confirm the trend that influencers primarily emphasize the benefits of the advertised dietary supplements without sufficiently pointing out the potential risks of taking them. On average, the posts on the 105 dietary supplements analyzed contained 148 words each. An average of 148 words is too short to give the user all the important information about the dietary supplement being advertised and to promote the dietary supplement at the same time.

Although information on adverse effects, drug interactions, and contraindications is not mandatory, it can represent important health-relevant information for the consumer. For example, a high-dose vitamin K supplement could limit the effect of a vitamin K-antagonist and thus lead to significant health risks for the consumer. Both manufacturers and influencers have a responsibility to consumers to inform them about potential risks. Influencers who reach a large number of people with their range of followers should act as role models and provide important health information. To convey all relevant health information about dietary supplements, posts on Instagram must be of an appropriate length—an average of 148 words per post is not enough. Regulatory frameworks for the advertising of dietary supplements, especially on Instagram, could help inform consumers not only about the potential benefits but also about the risks of dietary supplements. Overall, it would make sense to make information on adverse effects, drug interactions and contraindications mandatory in the Nahrungsergänzungsmittelverordnung (NemV) to avert potential health risks for consumers and protect their health.

### Discount codes

Influencers used discount codes to offer their followers the advertised product at a lower price and thus convince them to buy the product. Of the 105 dietary supplements analyzed, 63% (66) were advertised with a discount code. 37% of the dietary supplements (39) did not contain a discount code. A graphical representation of the results can be found in Figure S1 in the supplementary figures.

On average, the dietary supplements advertised were offered at a discount of 8.5%, while the median discount was 10%. The most frequently used discount code was a 10% discount code (39 preparations; 37%). The smallest discount code (after “no discount code”) was a 5% discount code (4 preparations; 4%). The largest discount code was a 40% discount code (1 preparation; 1%).

The use of discount codes by influencers is a targeted marketing strategy aimed at increasing the perceived attractiveness of the products and motivating potential buyers to purchase the dietary supplements. Discount codes offer consumers a direct financial incentive to buy the advertised product, which can lower the inhibition threshold for purchase. This is particularly relevant in the dietary supplement market, as the pricing of products is highly variable and often lacks transparency.

The true costs of dietary supplements are obscured using discount codes, making it difficult for consumers to assess the true value of the products. This lack of transparency could lead to consumers being tempted to buy the products even though there may be no actual need for the nutrients advertised.

Mandatory disclosure of the original price as well as the final price after the discount code has been applied could help consumers to make more informed decisions. Therefore, discount codes should be considered carefully, and it should be checked in advance how expensive the dietary supplement actually is and what benefit it has for the consumer.

### Suggestiveness of the product names in relation to a false or exaggerated effect of the preparation

Preparations were declared to be positively suggestive if their product name promised a false or exaggerated positive effect. For example, preparations with the names “Zell Boost,” “Flora Zauber,” or “Ah-Mazing Hair Vitamin” were rated as positively suggestive. Product names such as “Vitamin B12,” “Vitamin C Depot,” or “Zinc+Selenium” were rated as non-suggestive.

Of all preparations (105), the product names of 59 preparations (56%) were suggestive. The product names of the remaining 46 preparations (44%) were not suggestive. A graphical representation of the present results can be found in Figure S2 in the supplementary figures.

Of all combination products (81), 65% of the product names (53) were positively suggestive. Only one third of the product names of the combination preparations (28 preparations; 35%) did not suggest a false or exaggerated effect. The product names of the monopreparations (24) were not suggestive in 75% (18) of cases. In most cases, the active ingredient contained in the monopreparations was also included in the product name and thus suggested the effect of the corresponding active ingredient. 25% of the product names of the monopreparations (6) were positively suggestive.

The suggestive names of the preparations (59 preparations; 56%) can mislead consumers by raising unrealistic expectations about the effect of the preparations. In this way, consumers are lured into buying these preparations. This can lead to a consumer taking a product that is not necessary or even taking the wrong product. Purchasing decisions should be based on fact-based information and not on misleading product names. There should be a consistent regulation on the labeling of preparations that makes the active ingredients in the preparation clearly visible. This is the only way to create the necessary transparency for consumers.

### Promises of effectiveness from influencers

In addition to discount codes, influencers used promises of efficacy to convince consumers of the advertised dietary supplement. In this study, the influencers made a promise of efficacy for almost half of all dietary supplements advertised (50 preparations; 48%). A graphical representation of these results can be found in Figure S3 in the supplementary figures.

“With ‘Zell Boost’ you set the important basis for your longevity routine! I could already see positive changes in my skin after just one week and it is getting better every day. Simply fresh and radiant.” or “I ordered these drops mainly for my husband. Since he has been taking them daily, he feels better and fitter. He is no longer as tired as before.” were impressive quotes from influencers about the effects that consumers should experience by taking the dietary supplement.

Instagram’s Community Guidelines explicitly state that the dissemination of false messages that could increase the risk of violence or physical harm is not permitted. This also includes false claims that are potentially misleading and could lead to harm (Instagram). For example, the following quote from an influencer should be viewed very critically: “Spirulina slows down ageing processes, strengthens the immune system and thus protects against viral infections and cancer.”

Influencers also try to persuade consumers to buy the product with promises of effectiveness. Influencers mislead consumers with unobjective and exaggerated promises. Consumers can develop unrealistic expectations of the products and potentially take a risk by taking them. Influencers have a responsibility to their followers and should instead provide information about the actual effects of the dietary supplements. There should be stricter controls on Instagram to punish and prevent obviously misleading claims of efficacy. This can help to protect consumers from dangerous misinformation and potential risks.

## Limitations

The results represent a sample of dietary supplements from the wide range available on Instagram. Influencers around the world advertise new dietary supplements every day. As new dietary supplements with different active ingredient compositions are constantly coming onto the market, it is impossible to include all dietary supplements currently advertised on Instagram in the analysis.

The information on the dietary supplements comes from the product descriptions on the manufacturers’ websites. Influencers can subsequently edit their posts on Instagram and adapt them to the reactions of their subscribers and advertising partners—much of the information in the posts is therefore only a snapshot. The number of comments, followers, and likes can also be manipulated.

The ULs published by the EFSA provide indications of toxic effects if these values are exceeded. However, UL values are only defined for seven of the 13 vitamins and nine of the 17 minerals. There is insufficient data available for the remaining vitamins and minerals. The literature confirms toxic effects when individual active ingredients are overdosed, but no clear threshold can be defined above which toxic effects always occur.

## Conclusions

Dietary supplements advertised by German influencers on Instagram have significant shortcomings in terms of dosage overdoses and inadequate labeling of risks. Thus, Instagram does not constitute a suitable platform to obtain reliable information on dietary supplements. This is of great concern since Instagram is very popular.

Most of the preparations, mainly manufactured in Germany (90%) and partly in other EU countries (10%), contained several active ingredients, especially vitamins and minerals. These often exceeded the recommended reference values of the Bundesinstitut für Risikobewertung (BfR) and the daily reference amounts specified in the Verordnung (EU) Nr. 1169/2011. Some preparations even exceeded the tolerable upper intake level (UL), which poses a risk of toxic effects and a threat to consumer safety.

Although manufacturers usually provide user-friendly dosage information and mandatory warnings, this information was almost completely missing from the influencers’ posts, even though it is required by law. Without this information, consumers cannot assess whether the dosages correspond to their actual needs, which increases the risk of overdose and associated toxic effects.

The manufacturers only provided information on adverse effects, drug interactions, or contraindications for around one in five products. Almost without exception, such information was entirely missing from influencers´posts. This is particularly worrying as combination preparations with several active ingredients were advertised, which increases the risk of interactions, especially for older consumers with polypharmacy.

Influencers have a special responsibility towards their subscribers due to their large range of followers. However, instead of educating about the potential risks of supplementation, many of them used discount codes and exaggerated promises of efficacy to market the dietary supplements. The focus is obviously on capitalization, not on protecting consumers who are misled by suggestive dietary supplement names. Legal action is urgently required to counteract disinformation by influencers on social media channels.

For these reasons, it will make sense to label dietary supplements if they exceed the recommended reference values, e.g., those of the BfR. The marketing of dietary supplements that reach or exceed the UL should be strictly regulated to prevent toxic effects and protect the consumer. It would also be advisable to replace the mandatory dosage information, whose reference values date back to 2011 (Verordnung (EU) Nr. 1169/2011), with a more up-to-date version, such as that of the BfR from 2021.

In view of the toxic consequences of an overdose of vitamins or minerals described in the literature, supplementation with dietary supplements should be carefully evaluated and ideally only undertaken after consultation a doctor. It is important that vitamin and mineral doses do not reach the UL.

## Future studies

Future research should aim to test the insights gained in this analysis on the promotion of potentially overdosed dietary supplements by influencers on Instagram in other countries to assess the generalizability of the results pertinent to Germany. Similarly, it will be important to analyze the presentation of dietary supplements on other social media channels.

Further research is needed to identify missing tolerable upper intake levels (ULs) for vitamins and minerals. For almost half of the vitamins (6 out of 13) and minerals (8 out of 17) no UL values were set in the 2018 EFSA report “Overview on Tolerable Upper Intake Levels as derived by the Scientific Committee on Food (SCF) and the EFSA Panel on Dietetic Products, Nutrition and Allergies (NDA)” due to a lack of sufficient data. The determination of these missing ULs is of great importance about consumer protection, as high-dose dietary supplements of these vitamins and minerals are already available on the market.

## Supplementary Information

Below is the link to the electronic supplementary material.

Supplementary file1 (DOC 171 KB)

## Data Availability

All source data for this work (or generated in this study) are available upon reasonable request.

## Abbreviations

AMG: Arzneimittelgesetz (Medical Products Act)
AMPreisV: Arzneimittelpreisverordnung (Pharmaceuticals Pricing Regulation)
BfR: Bundesinstitut für Risikobewertung (Federal Institute for Risk Assessment)
BVL: Bundesamt für Verbraucherschutz und Lebensmittelsicherheit (Federal Office of Consumer Protection and Food Safety)
DGE: Deutsche Gesellschaft für Ernährung (German Society for Nutrition)
EFSA: European Food Safety Authority
LFGB: Lebensmittel- und Futtermittelgesetzbuch (German Food and Feed Code)
NEM: Nahrungsergänzungsmittel (dietary supplements)
NemV: Verordnung über Nahrungsergänzungsmittel (Dietary Supplements Ordinance)
SCF: Scientific Committee on Food
TBK: Tagesbehandlungskosten (daily therapy costs (DTC))
UL: Tolerable upper intake level
