# Supplementary material for: Disinformation on dietary supplements by German influencers on Instagram
Source: Naunyn Schmiedebergs Arch Pharmacol. 2024 Nov 25;398(5):5629–47. doi: 10.1007/s00210-024-03616-4 (PMC11985574; doi:10.1007/s00210-024-03616-4)

**Supplemental figures - Disinformation on dietary supplements by German influencers on Instagram**

Jan-Niklas Ricke · Roland Seifert

**Figure S1** Distribution of discount codes offered by influencers as a bar chart


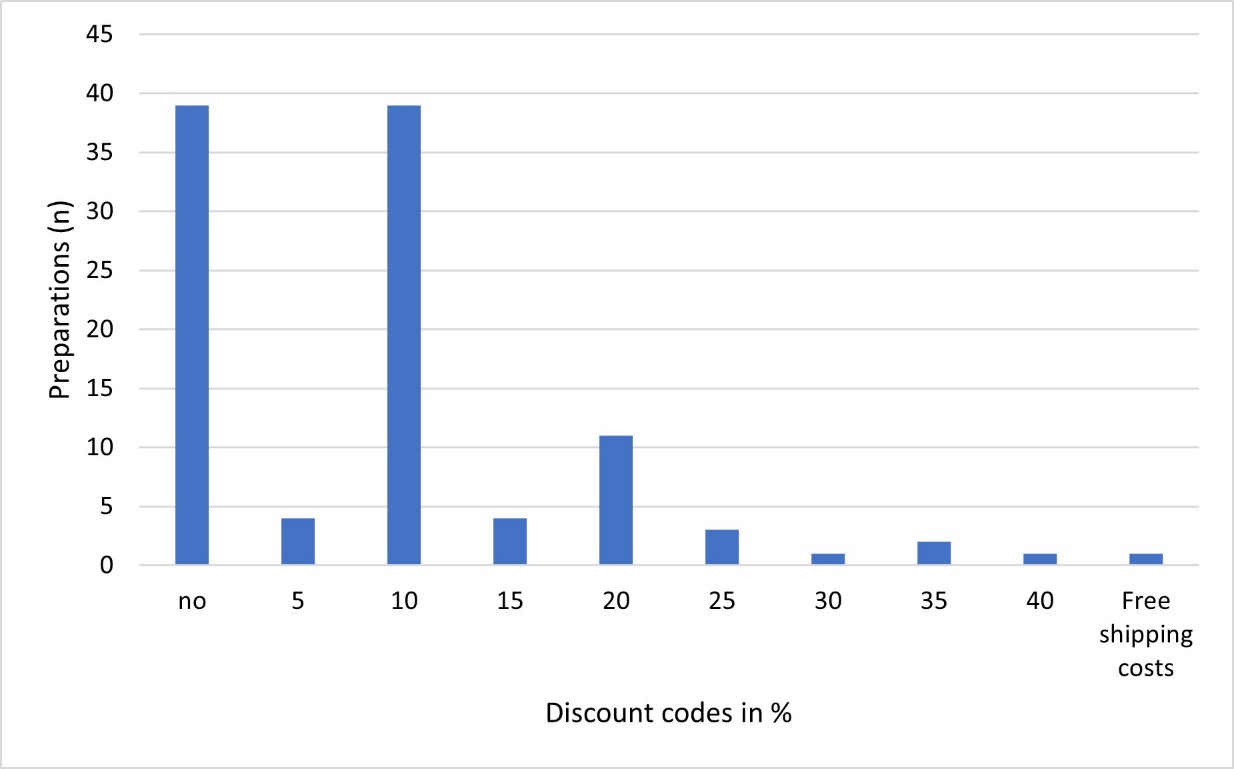


**Figure S2** Representation of the frequency of suggestiveness of the preparations as a pie chart, whereby the proportion of preparations with suggestiveness is coloured blue and the proportion of preparations without suggestiveness is coloured orange


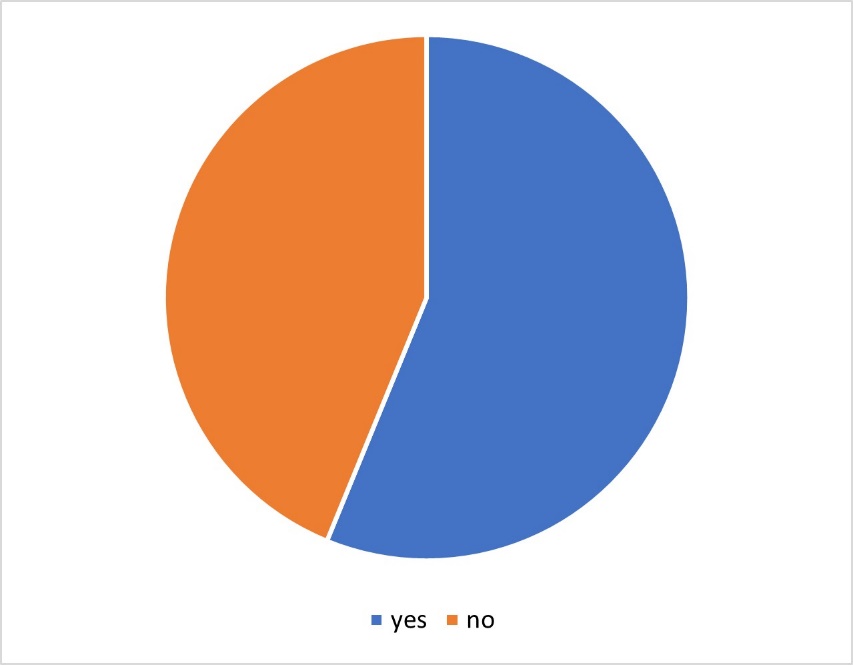


**Figure S3** Presentation of the frequency of a promise of effectiveness in the posts as a pie chart, with the proportion of posts with a promise of effectiveness coloured blue and the proportion of posts without a promise of effectiveness coloured orange


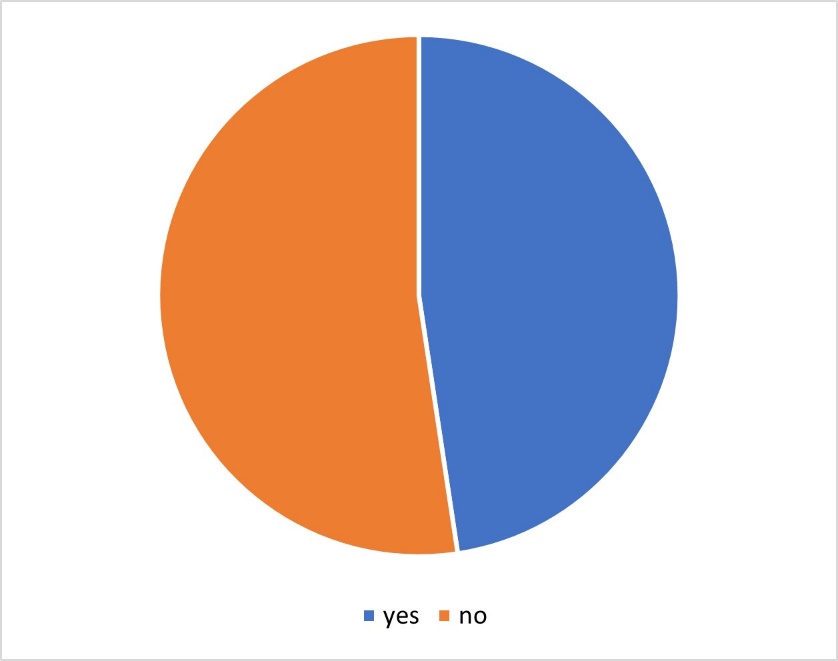

Supplement: Supplementary file 1 — Supplementary file1 (DOC 171 KB) [file 210_2024_3616_MOESM1_ESM.docx]
